# Human limb skeletal muscle wasting and architectural remodeling during five to ten days intubation and ventilation in critical care – an observational study using ultrasound

**DOI:** 10.1186/s12871-016-0269-z

**Published:** 2016-11-29

**Authors:** Peter Turton, Richard Hay, Jonathon Taylor, Jamie McPhee, Ingeborg Welters

**Affiliations:** 1 Intensive Care Unit, Royal Liverpool University Hospital, Prescot Street, Liverpool, L7 8XP UK; 2 Intensive Care Unit, Warrington General Hospital, Lovely Lane, Warrington, WA5 1QG UK; 3 School of Healthcare Science, Manchester Metropolitan University, Chester Street, Manchester, M15 5GD UK; 4 Institute of Aging and Chronic Disease, University of Liverpool, 4th floor, UCD Building, Daulby Streey, Liverpool, L69 3GA UK

**Keywords:** Muscle wasting, Weakness, Critical care, Ultrasound, Muscle architecture, Pennation angle

## Abstract

**Background:**

Critically ill patients frequently suffer muscle weakness whilst in critical care. Ultrasound can reliably track loss of muscle size, but also quantifies the arrangement of the muscle fascicles, known as the muscle architecture. We sought to measure both pennation angle and fascicle length, as well as tracking changes in muscle thickness in a population of critically ill patients.

**Methods:**

On days 1, 5 and 10 after admission to critical care, muscle thickness was measured in ventilated critically ill patients using bedside ultrasound. Elbow flexor compartment, medial head of gastrocnemius and vastus lateralis muscle were investigated. In the lower limb, we determined the pennation angle to derive the fascicle length.

**Results:**

We recruited and scanned 22 patients on day 1 after admission to critical care, 16 were re-scanned on day 5 and 9 on day 10. We found no changes to the size of the elbow flexor compartment over 10 days of admission. In the gastrocnemius, there were no significant changes to muscle thickness or pennation angle over 5 or 10 days. In the vastus lateralis, we found significant losses in both muscle thickness and pennation angle on day 5, but found that fascicle length is unchanged. Loss of muscle on day 5 was related to decreases in pennation angle. In both lower limb muscles, a positive relationship was observed between the pennation angle on day 1, and the percentage of angle lost by days 5 and 10.

**Discussion:**

Muscle loss in critically ill patients preferentially affects the lower limb, possibly due to the lower limb becoming prone to disuse atrophy. Muscle architecture of the thigh changes in the first 5 days of admission, in particular, we have demonstrated a correlation between muscle thickness and pennation angle. It is hypothesised that weakness in the lower limb occurs through loss of force generation via a reduced pennation angle.

**Conclusion:**

Using ultrasound, we have been able to demonstrate that muscle thickness and architecture of vastus lateralis undergo rapid changes during the early phase of admission to a critical care environment.

## Background

Intensive Care Unit Acquired Weakness (ICUAW) is a common consequence of prolonged stay in the intensive care setting. Generalised muscle weakness due to muscle wasting is a key feature of the disease [1]. As a result, survivors of intensive care admission have difficulty with mobility and require complex rehabilitation [2, 3].

Previous work on ICUAW has identified many inter-related factors that put patients at risk of muscle wasting, including increased protein degradation, the use of steroids, high levels of circulating pro-inflammatory mediators and the presence of multi organ failure [4, 5]. The prolonged immobility of intubated and ventilated patients has also been suggested to be a significant risk factor [6].

Ultrasound is increasingly being used to assess both cross sectional area and muscle thickness at the bedside of the patient, and is a useful tool in monitoring changes in muscle size over time [7]. Furthermore, when predicting adverse outcome in the intensive care environment, ultrasound assessment of the rectus femoris muscle has been shown to compare favourably with measures of frailty [8]. In studies performed in healthy subjects, measurements of muscle thickness and fascicle arrangement of the muscle using ultrasound are reproducible and are not dependent on age [9]. In the intensive care population, significant reductions in the cross sectional area of the rectus femoris muscle over a 10 day admission period have been demonstrated [10]. Muscle thickness of the quadriceps femoris has also been shown to decrease over 28 days, with muscle thickness also being negatively correlated with the length of stay [11].

As well as muscle thickness and cross sectional area, ultrasound can also be used to visualise the arrangement of the fascicles within the muscle, which informs about both the pennation angle and the length of the fascicle.

It has been shown in the triceps brachii that muscle thickness is positively correlated with pennation angle [12] and further, pennation angle is positively correlated with the cross sectional area of vastus lateralis [13] and rectus femoris [14]. Muscle force has been suggested to increase with increasing pennation angle, up to a limit of 45 degrees [15]. The same paper found that in healthy subjects, resistance based strength training was associated with an increased cross sectional area, pennation angle and contractile strength, suggesting an inter-relation between muscle architecture variables and force generation. However, pennation angle has never been used to assess muscle wasting in critically ill patients.

In this study, we aimed to establish changes in pennation angle and fascicle length as markers of altered fascicle arrangement in critical illness. We furthermore wanted to compare muscle wasting in areas of the upper and the lower limb by assessing muscle thickness of the vastus lateralis muscle, the medial head of gastrocnemius muscle and the elbow flexor compartment in ventilated patients.

## Methods

### Participants

The study received ethical approval from the Local Research Ethics Committee (UK NHS Health Research Authority, Wales Research Ethics Committee 4 (REC 4) Wrexham, Wales, United Kingdom, approval number 13/WA/0111). Patients were recruited from the Intensive Care Units of the Royal Liverpool University Hospital, or Warrington District General Hospital over a 1 year study period. Informed assent was obtained from the patients’ next of kin, and where possible, retrospective consent was sought when the patients had regained capacity.

Patients who were greater than 18 years of age who were assented within 24 h of being intubated and admitted to the participating intensive care units were included in the study. Exclusion criteria included being under 18 years of age pregnancy, trauma to either the right lower or right upper limbs, history of neurological, neuromuscular or muscular wasting diseases, rhabdomyolysis, vascular insufficiency or amputation of the right upper or lower limbs, and prolonged immobility prior to admission to intensive care.

### Ultrasound scanning

On day 1 of imaging, all patients were intubated, ventilated and sedated. Imaging on subsequent days was performed in patients who were still intubated and ventilated, but may not have been sedated, according to their clinical progress. None of the patients were given continuous muscle relaxant infusions on the day of scanning.

**Fig. 1:**
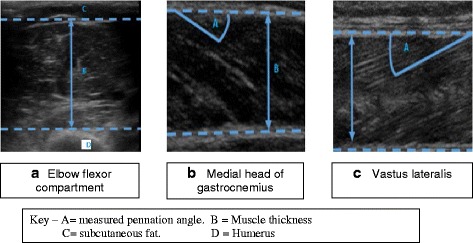
Representative images of elbow flexor compartment (**a**), gastrocnemius (**b**) and vastus lateralis (**c**).

Patients had ultrasound scans of the right elbow flexor compartment (Fig. 1a), right medial head of gastrocnemius (Fig. 1b) and right vastus lateralis (Fig. 1c) muscles were performed within 24 h of intubation. Ultrasound scans were undertaken with B-mode ultrasonography, using a linear array probe. One of three trained operators performed the ultrasound scans, and it was ensured that when an operator scanned a participant, the same researcher performed the repeat scans on days 5 and 10. Patients were positioned in the supine anatomical position for ultrasound scanning, using a method previously described in a study of healthy volunteers undergoing prolonged bedrest [16].

Imaging of the right elbow flexor compartment was undertaken with the patient sat up at 45 degrees and the limbs positioned in the anatomical position, ensuring there was no flexion at the elbow. The ultrasound transducer was placed 5 cm proximal to the line connecting both the medial and lateral epicondyles, above the base of the ante cubical triangle and was positioned perpendicular to the plane of the muscle, to image the muscle thickness from the edge of muscle down to the tip of the humerus. Scanning in plane with the muscle was avoided, as in the upper limb, fascicles run parallel with the aponeuroses, making calculation of fascicle length inaccurate.

Muscle thickness measurement was made by firstly measuring the overall width seen on the image, from the top of the muscle down to the bottom of the image. Secondly, the distance from the tip of the humerus to the bottom of the image was measured and subtracted from the overall thickness to give the thickness of the muscle from humerus to the superior muscle edge.

Six images were taken per patient, the highest and lowest muscle width was removed, and the average of the four remaining images was used to give the final measurement for each patient.

For scanning of the vastus lateralis, patients remained sat at 45 degrees, but with the lower limb flat to the bed. The limb was then externally rotated to ensure the probe handle could be placed parallel to the ground. The probe was placed 10 cm proximal to the lateral condyle of the femur, in plane with the muscle to image both the superficial and deep aponeuroses of the vastus lateralis, and the muscle fascicles within the muscle. Imaging of the medial gastrocnemius was achieved by flexion at the knee to ensure the thigh was at 90 degrees to the tibia, with the foot placed flat onto the patient’s bed. This is different to method used in healthy volunteers [16], but due to the inflatable mattresses used, this method exposes the medial head to enable imaging, again with the probe handle parallel to the ground. The probe was again placed in plane with the muscle, at the widest point in the muscle belly. Depth of scanning was altered until both the superficial and deep aponeuroses were visible, along with the fascicles within the muscle.

In both lower limb muscles, six images were taken. Muscle thickness (MT) was measured at the widest point in each image, from the superficial to the deep aponeuroses. Pennation angle (PA) was measured by measuring the angle in degrees between the fascicle and the superficial aponeurosis. This method measures the fascicle closest to the widest point in the muscle to minimise variation in pennation angle in just one muscle [17]. Fascicle length (FL) was derived from pennation angle and muscle thickness as described before [18, 19]^,^. Briefly, the following formula was used to derive fascicle length:

$$ \mathrm{F}\mathrm{L} = \mathrm{M}\mathrm{T}\ /\ \left( \sin\ \mathrm{P}\mathrm{A}\right) $$

Muscle thickness was measured in pixels initially, before converting into centimetres, as each image had a scale of known depth running down the right hand side of the image, which always consisted of 380 pixels in depth. To prevent compression of the muscle during imaging, the ultrasound probe was coated with a thick layer of a water-based gel.

Once all six images were taken from each muscle, the images with the longest and shortest fascicle length were removed, and the average muscle width, pennation angle and fascicle length were calculated from the remaining four images. Image measurement was undertaken using ImageJ software (version 1.47, National Institutes of Health, USA).

Imaging was repeated on days 5 and 10, provided the patient remained intubated. Patients were not scanned again if they were extubated or had died prior to their next scheduled scan.

### Statistics

Muscle thickness, pennation angle and fascicle length have been assessed using Friedman’s test. Pairwise Friedman’s tests were performed where results were significant (see Table 3). Significance has also been tested by constructing 95 % confidence intervals for absolute changes to muscle thickness and pennation angle at both days 5 and 10, with intervals not passing through zero considered to be statistically significant.

Where correlations were tested, Pearson’s product moment correlation coefficient was used, with a *p*-value of less than 0.05 being considered statistically significant. Changes in demographic data over time were assessed used a one-way ANOVA, with a Bonferroni correction applied where significant differences were found. Data was analysed using SPSS version 21 for Windows (IBM, USA).

## Results

### Patient demographics

**Fig. 2:**
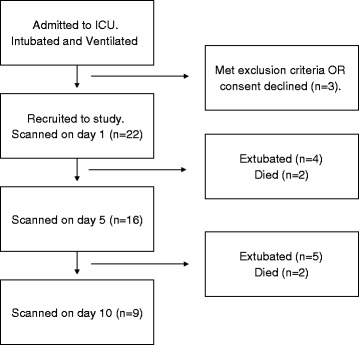
Study recruitment flow diagram

Twenty two patients were recruited into the study and imaged on day 1. Of these, 16 patients were scanned on day 5, and 9 were scanned on day 10. Patients who were not scanned on day 5 or 10 were either successfully extubated or died before their next scan (Fig. 2). Table 1 summarises the major patient demographics. There were no significant changes to age, weight, height or body mass index (BMI) during the study period. All patients met the criteria for SIRS, but not all had a diagnosis of sepsis (Table 2).

**Table 1:** Patient characteristics

	Day 1	Day 5	Day 10	*P*-value
Sample Size	22	16	9	---
Males, n (%)	19 (86.3)*	13 (81.3)	7 (77.7)	* < 0.001
Age, [years] (Mean ± SD)	59.77 ± 16.74	63.12 ± 16.44	58.66 ± 19.95	0.78
Height, [metres] (Mean ± SD)	1.72 ± 0.13	1.68 ± 0.13	1.71 ± 0.13	0.78
Weight, [kg] (Mean ± SD)	88.50 ± 29.19	82.95 ± 27.31	75.66 ± 24.58	0.54
Body Mass Index, (Mean ± SD)	30.75 ± 12.50	30.99 ± 13.69	25.73 ± 7.40	0.61

Data tested with one-way ANOVA with Bonferroni correction applied. Statistically significant *p*-values (*p*<0.05) have been marked with *. Despite patients withdrawing throughout the study period, there were no significant changes to group’s demographic variables. The number of males compared to females on day 1 was compared using the binomial test

**Table 2:** Patient characteristics by presenting diagnosis

Catergory of diagnosis	Number of patients, N, (%)
Sepsis	11 (50 %)
Post surgery	4 (18 %)
Post cardiac arrest	4 (18 %)
Overdose	3 (9 %)

All patients met the criteria for Systemic Inflammatory Response Syndrome (SIRS)

### Changes in muscle thickness

A summary of measurements in all three muscles (Vastus lateralis muscle, medial head of gastrocnemius muscle, elbow flexor compartment) is presented in Table 3. Baseline measurements were performed on day 1. A significant correlation between muscle thickness in the upper and lower limb was demonstrated (Table 4).

**Table 3:** Changes in measured variables in the elbow flexor compartment, gastrocnemius and vastus lateralis muscles

	Day 1 (*n* = 22)	Day 5 (*n* = 16)	Day 10 (*n* = 9)	*P*-value
MT – Elbow flexor compartment, [cm] (median ± IQR)	3.20 ± 0.58	3.30 ± 0.87	2.98 ± 0.83	0.62
MT – Gastrocnemius, [cm] (median ± IQR)	1.29 ± 0.60	1.34 ± 0.43	1.14 ± 0.65	0.72
MT – Vastus lateralis [cm] (median ± IQR)	1.53 ± 0.77	1.40 ± 0.46	1.18 ± 0.36	0.002*
FL – Gastrocnemius [cm] (median ± IQR)	3.99 ± 1.84	3.69 ± 1.37	3.43 ± 2.12	0.90
FL – Vastus lateralis [cm] (median ± IQR)	8.1 ± 3.06	8.45 ± 4.55	7.85 ± 5.89	0.89
PA – Gastrocnemius [degrees] (median ± IQR)	20.93 ± 6.41	19.82 ± 5.65	17.5 ± 3.91	0.37
PA – Vastus lateralis [degrees] (median ± IQR)	11.09 ± 4.88	9.86 ± 3.69	8.03 ± 3.86	0.018*

*MT* Muscle thickness, *FL* Fascicle length, *PA* Pennation angle

Significance tested with Friedman’s test, followed by pairwise Friedman’s tests where *p* < 0.05. Stastically significant *p*-values have been marked with *. Muscle thickness and pennation angle of the vastus lateralis muscle significantly decreased over both 5 and 10 days

**Table 4:** Comparison of muscle thickness between all three muscles on day 1

Muscle 1	Muscle 2	Correlation coefficient, r	*P*-value
Elbow flexor compartment	Vastus lateralis	0.50	0.01^*^
Elbow flexor compartment	Medial head of gastrocnemius	0.54	0.006^*^
Vastus lateralis	Medial head of gastrocnemius	0.60	0.002^*^

Pearson’s product–moment correlation coefficient, *p < 0.05

### Elbow flexor compartment (Fig. 1a)

On day 1, there was a significant negative correlation between age and muscle thickness (*r* = −0.39, *p* = 0.03, *n* = 22). Height and weight were both significantly positively correlated with muscle thickness (height *r* = 0.41, *p* = 0.04; weight *r* = 0.57, *p* = 0.006).

There were no significant changes in muscle thickness in the elbow flexor compartment on either day 5 or day 10, when compared to day 1. Mean change in muscle thickness on day 5 was 2.62 % (95 % CI −2.12 to 7.36 %, *p* = 0.26) and on day 10 was 3.79 % (95 % CI: −15.70 to 23.28 %, *p* = 0.66). Age, weight, height and APACHE II score on admission did not correlate with absolute or relative change in muscle thickness.

### Medial head of gastrocnemius muscle (Fig. 1b)

There were no significant changes in muscle thickness of the medial head of the gastrocnemius muscle on either day 5 or 10. On day 5, mean change in muscle thickness was −5.08 % (95 % CI: −14.03 % to 3.88 %, *p* = 0.25), and on day 10, the mean change in muscle thickness was −15.69 % (95 % CI: −43.43 % to 12.05 %, *p* = 0.23).

Muscle thickness on day 1 was negatively correlated with absolute change in muscle thickness on day 5 (*r* = −0.53, *p* = 0.02) and day 10 (*r* = −0.662, *p* = 0.03).

### Vastus lateralis (Fig. 1c)

In the vastus lateralis muscle, age was negatively associated with muscle thickness (*r* = −0.54, *p* = 0.007) and fascicle length (*r* = −0.53, *p* = 0.007). Further, patient weight was positively associated with muscle thickness (*r* = 0.41, *p* = 0.04).

There were significant losses of both muscle thickness over both 5 and 10 days; a mean change of −10.66 % (95 % CI: −17.48 to −3.83 %, *p* = 0.005) was seen on day 5, a mean change of −28.81 % (95 % CI: −37.39 to −20.23 %, *p* < 0.0001) on day 10, see Fig. 4a.

Muscle thickness on day 1 was not significantly correlated with absolute change in muscle thickness on day 5 (*r* = −0.43, *p* = 0.05) but this correlation was observed by day 10 (*r* = −0.770, *p* = 008).

### Changes in lower limb muscle architecture

#### Medial head of gastrocnemius muscle

In both the medial head of gastrocnemius and vastus lateralis muscles, no relationship was observed between the changes in muscle architecture and age, weight, height, BMI or APACHE II score.

On day 1, muscle thickness of gastrocnemius muscle was positively correlated with pennation angle (*r* = 0.41, *p* = 0.03, *n* = 21), and positively correlated with fascicle length on day 1 (*r* = 0.60, *p* = 0.002). Pennation angle was negatively associated with fascicle length on day 1 (*r* = −0.43, *p* = 0.028).

On day 5, mean change in pennation angle was 0.92 % (95 % CI: −9.02 to 7.17 %, *p* = 0.81), and mean change in fascicle length was 2.47 % (95 % CI: −13.11 to 8.15 %, *p* = 0.63). On day 10, mean change in pennation angle was −6.73 % (95 % CI: −23.13 to 9.66 %, *p* = 0.37) and mean change in fascicle length was −9.89 % (95 % CI: −31.45 to 11.67 %, *p* = 0.32).

**Fig. 3:**
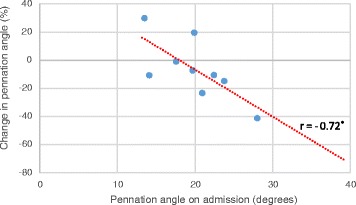
Pennation angle of gastrocnemius on day 1 and change in pennation angle on day 10 Correlation tested using Pearson’s product–moment correlation coefficient. A significant negative relationship was observed between pennation angle on admission and the change in pennation angle size on day 10

Pennation angle on day 1 was significantly negatively correlated with percentage change on day 5 (*r* = −0.71, *p* = 0.009, *n* = 16) and day 10 (*r* = −0.72, *p* = 0.014, *n* = 9, see Fig. 3).

### Vastus lateralis muscle

**Fig. 4:**
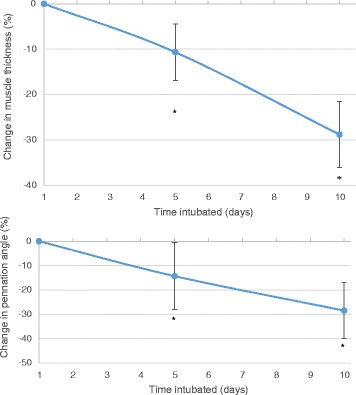
Changes to muscle thickness and pennation angle in vastus lateralis muscle during critical care admission. Top panel: Change in muscle thickness. Error bars are 95 % confidence intervals. Bottom panel: Change in pennation angle. Error bars are 95 % confidence intervals

For pennation angle, a mean change of −14.31 and −28.47 % was seen on days 5 and 10 respectively (Day 5 95 % CI: −25.53 to −3.09, *p* = 0.006; Day 10: −41.98 to −14.95, *p* = 0.001, see Fig. 4b). There were no significant changes to fascicle length on either day.

**Fig. 5:**
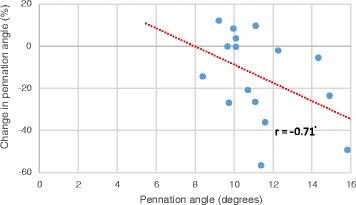
Pennation angle of vastus lateralis on day 1 and change in angle on day 5. Correlation tested using Pearson’s product–moment correlation coefficient. A significant correlation was noted between the pennation angle on admission, and the percentage change in pennation angle on day 5

Pennation angle on day 1 was significantly negatively associated with mean change in angle on day 5 (*r* = −0.43, *p* = 0.04, see Fig. 5).

**Fig. 6:**
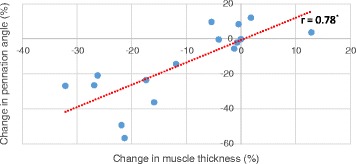
Changes in muscle thickness and pennation angle on day 5 in the vastus lateralis muscle. Correlation tested using Pearson’s product–moment correlation coefficient. On day 5, a significant relationship was observed between the change in muscle thickness and the change in pennation angle

Mean change in muscle width on day 5 was significantly positively correlated with mean change in pennation angle on day 5 (*r* = 0.78, *p* < 0.001, see Fig. 6).

## Discussion

In this study we have demonstrated a loss in muscle thickness and a decrease in pennation angle in the vastus lateralis. These results support previous studies demonstrating that admission to intensive care causes a reduction in muscle size in the thigh [10, 11], with of loss of muscle thickness in the vastus lateralis muscle occurring within the first 5 days of admission. In our study, muscle wasting occurred earlier than reported in the literature: a previous study found that the cross sectional area of the rectus femoris muscle significantly decreased in the first 7 days of admission, whilst showing no changes in the first 3 days [10]. Further loss of muscle thickness has also been related with loss of muscle function on discharge from critical care [20].

In our study, this loss of muscle thickness in the thigh is coupled with a significant reduction in the pennation angle of the muscle fascicles, but maintaining fascicle length. The decline in pennation angle continues until day 10 post-intubation. In the medial gastrocnemius we have shown similar trends towards loss of both muscle thickness and pennation angle in the first 96 h, however these results did not reach statistical significance. Large pennation angles are able to pack more contractile components in parallel into a certain volume, and can generate greater force up to angles of 45 degrees [15, 21]. If loss of angle leads to a loss of force generation, then this could help to explain the weakness patients experience on being discharged from critical care. We further demonstrated that in both muscles, vastus lateralis and gastrocnemius, muscle thickness and pennation angle are related and support previous studies that thicker muscles could generate greater force by having larger pennation angles, and consequently, larger cross sectional areas [15] There was also a relationship in both muscles between the pennation angle on day 1, and how much of that angle was lost by day 5, suggesting that patients who had a larger pennation angle on day 1 lost a greater percentage of their pennation angle than patients who were admitted with smaller pennation angles. This relationship also held for muscle thickness, with muscle thickness decreasing to a greater degree in thicker muscles. This may indicate that muscles with larger pennation angles also have greater muscle thickness by having greater numbers of sarcomeres in parallel to the direction of the fascicle. It may well be that these parallel sarcomeres are lost first, causing loss of pennation angle, and consequently loss of muscle thickness; a recent review article has also suggested that physical training may increase muscle cross sectional area by increasing parallel addition of sacromeres, and are lost in the same way after a period of disuse [22]. The lack of reduction in fascicle length may suggest that serial sarcomeres are preserved.

Age-related deterioration of the pennation angle of both the vastus lateralis muscle [9] and medial gastrocnemius muscle [23] have previously been described, as well an age-related loss of muscle thickness [24]. To our knowledge, this is this first paper that describes how the muscle architecture of pennate muscles changes due to immobility through intubation and ventilation. This addresses previous criticism of a recent literature review describing that although ultrasound is used to measure muscle thickness and cross sectional area, the changes to muscle architecture have not been described yet [25].

Interestingly, the changes we describe above were strictly limited to the lower limb and could not be reproduced in elbow flexor compartment. The size of the elbow flexor compartment remained unchanged throughout the first 10 days of admission. These results are in line with a previous study in healthy volunteers undergoing a five week period of bed rest that had shown that muscle thickness decreased in the both the vastus lateralis and medial gastrocnemius, but not in the biceps brachii or tibialis anterior [16]. Our findings support the theory that in healthy and critically ill individuals, non-weight bearing muscles are less susceptible to wasting, compared to muscles which have a daily load placed up on them through the action of walking or standing.

Due to its force- generating properties, pennation angle should be monitored in all interventions aimed at preventing muscle wasting in critical illness. Both early mobilisation [26] and neuromuscular electrical stimulation [27] have been shown to prevent muscle atrophy in critical care patients, although their effects on muscle architecture have not yet been described and needs to be elucidated in further studies.

Our study is limited by several factors, in particular the small sample size and the heterogeneity of the patient cohort. There were significantly more males recruited compared to females, suggesting our results may not be applied to both sexes. Our study was standardised so that the right upper and lower limbs where measured, however hand dominance was not established prior to starting, and is a further limitation.

All patients met the criteria for SIRS, however not all of them were septic at the time of ultrasound scanning. Further, measurement of inflammatory markers was not undertaken at the time of scanning, nor have any muscle biopsies been taken from these patients. A larger sample would be required to compare if patients with sepsis lose more muscle compared to non-septic individuals.

## Conclusion

Muscle architecture in the lower, but not in the upper limb is altered within the first five days of being intubated and mechanically ventilated, potentially reducing force generation and contributing to ICU acquired weakness. We were unable to demonstrate a change in the thickness of the muscles of the elbow flexor compartment suggesting weight bearing muscles are at greater risk of ICU acquired weakness. Monitoring of changes to muscle architecture may lead to early detection and better quantification of muscle loss compared to sole measurement of muscle thickness. Further prospective studies are required to determine if loss of pennation angle is found in the presence of a clinically diagnosed ICU acquired weakness. Interventional studies to preserve muscle function and architecture should include imaging of muscle architecture.

## Key messages

- Muscle wasting occurs rapidly in the intensive care environment, but appears to preferentially affect the lower limb.
- Muscle thickness and pennation angle are lost during long term intubation and ventilation, however fascicle length appears to remain constant.
- Loss of muscle thickness is related to loss pennation angle by day 5.
- Loss of pennation angle and muscle thickness by day 5 are related to their initial sizes on admission – large muscles appear to lose more by day 5.

## Abbreviations

FL: Fascicle length
ICUAW: Intensive Care Unit Acquired Weakness
MT: Muscle thickness
PA: Pennation angle
